# Supplementary material for: Autonomous platform for solution processing of electronic polymers
Source: Nat Commun. 2025 Feb 17;16:1498. doi: 10.1038/s41467-024-55655-3 (PMC11833048; doi:10.1038/s41467-024-55655-3)
Supplement: Supplementary file 1 — Supplementary Information [file 41467_2024_55655_MOESM1_ESM.pdf]

Supporting Information

Autonomous Platform for Solution Processing of Electronic Polymers

Chengshi Wang [1]†, Yeon-Ju Kim [1]†, Aikaterini Vriza [1]†, Rohit Batra [1,5]†, Arun Baskaran [1,6], Naisong Shan [2], Nan Li [2], Pierre Darancet [1], Logan Ward [3], Yuzi Liu [1], Maria K.Y. Chan [1], Subramanian K.R.S. Sankaranarayanan, [1,4], H. Christopher Fry, [1] C. Suzanne Miller [1], Henry Chan [1]*, Jie Xu [1,2]*

[1]*Nanoscience and Technology Division, Argonne National Laboratory, Lemont, IL 60439, USA.*

[2]*Pritzker School of Molecular Engineering, The University of Chicago, Chicago, IL 60637, USA.*

[3]*Data Science and Learning Division, Argonne National Laboratory, Lemont, IL 60439, USA*

[4]*Department of Mechanical and Industrial Engineering, University of Illinois, Chicago, Illinois 60607.*

†These authors contributed equally to this work. [5]*Present address: Department of Metallurgical and Materials Engineering, Indian Institute of Technology Madras, Chennai 600036, India.* [6]*Present address: Corning Incorporated, Corning, NY 14831, USA.*

*Corresponding authors. Email: xuj@anl.gov; hchan@anl.gov

The PDF file includes:

Supplementary Table 1: Summary of the experimental parameters. The ranges of these parameters are discretized evenly, giving 36 formulations, 90 processing conditions, and 288 post-processing conditions (a total of 933120 possible experimental conditions). 5

Supplementary Table 2. Number and Mixing ratios of post-processing solvents. 5

Supplementary Fig. 1. Automated workflow of our thin film experiment. The workflow includes steps in our experimental procedure (described in Section 2) and other additional steps specific to an automated workflow. The graphical user interface allows users to set the experimental parameters and initiate the running of a sample...6

Supplementary Fig. 2. Example images captured by the automated imaging system in Polybot. The images are color corrected using the gray card and processed by computer vision algorithms for film coverage analysis. 9

Supplementary Fig. 3. Measurement locations in a sample. The sample size is 2cm x 2cm. Red points mark the locations of four thickness characterizations and yellow points mark the locations of eight four-point probe measurements. 10

Supplementary Fig. 4. Automated thickness measurement station. The sample is rotated by a robotic gripper for measuring different sample locations. 11

Supplementary Fig. 5. Proof of ML model success. On average, the processing conditions identified by the ML model result in higher conductivity samples (blue circles). The average conductivity in the start from training data is around 300 S/cm. However, at the end of 45 test experiments, the avg. conductivity is 1075 S/cm (more than triple). This proves that the ML model successfully learned what processing conditions result in high conductivity films. After applying a non-monotonic fit method on the test data, it was observed that the resulting curve exhibits a distinct pattern: while it shows variation at the beginning, it eventually transitions into a linear pattern during the final iterations. As such the closed-loop experiment was terminated after reaching this consistent linear pattern. 12

Supplementary Fig. 6. Scatterplot showing the trade-off between average conductivity and average coverage for both the training and test data. Only the test datapoints are labelled in the plots and the champion candidates for further exploration are highlighted in yellow. 13

Supplementary Table 3. Actual values of the processing parameters for the three selected champion candidates. 14

Supplementary Fig. 7. Correlation matrices for conductivity (left) and coverage (right) on the final dataset acquired from the autonomous experiment. 14

Supplementary Table 4. Machine learning regression models for coverage prediction on the train data only. A random seed of 1 was used in all the cases to achieve repeatability. 16

Supplementary Fig. 8. a) Shapley feature importance showing the effect of the various parameters in coverage after fitting the best performing Random Forest model on the train data. We can clearly see

that the top three important features are the DMSO%, coating temperature with a negative impact to coverage and coating speed with a positive impact in coverage. b) Density mapping of the most important parameters that affect the coverage as observed. 16

Supplementary Table 5. Machine learning regression models for predicting conductivity using training data only. A random seed of 1 was used in all the cases to achieve repeatability. Given the uneven distribution of the target property, achieving predictive accuracy is challenging due to the significantly imbalanced data. 16

Supplementary Table 6. ML models for coverage predictions. For each model type, three separate models are trained using: training data only, test data only, and all data (training + test data). The random seed parameter was set to 1 for repeatability. 17

Supplementary Table 7. ML models for conductivity predictions. 20

Supplementary Fig. 9. Parity plots for the coverage and conductivity predictions. a) Predicted versus actual coverage after fitting the best performing model in the collected datapoints. b) Predicted versus actual average conductivity after fitting the best performing model in all the collected data points. ...20

Supplementary Fig. 10. Large PEDOT:PSS conductive thin films prepared using blade coating.....21

Supplementary Fig. 11. The working principle of cryo-EM on frozen polymer solution.The cryo-EM samples were prepared by a Thermo Scientific Vitrobot Mark IV System. The samples were loaded to a FISCHIONE 2550 cryo transfer tomography holder for imaging on a FEI Talos 200FX (S)TEM operated at 200kV. 21

Supplementary Fig. 12. GIWAXS 2D images of three PEDOT:PSS thin films. 22

Supplementary Fig. 13. Polarized ultraviolet − visible absorption spectra of blade-coated PEDOT:PSS film from a solution with 5 vol% EG, coated at 1 mm/s, 90℃, and post-processed with methanol/ethanol (4:6), coated at 1 mm/s, 60℃. The red (Para) curve was obtained when the film's coating direction was aligned with the polarizer axis, whereas the gray curve (Perp) was collected with the coating direction perpendicular to the polarizer axis. 22

Supplementary Fig. 14. S (2p) X-ray photoelectron spectra of spin-coated film from pristine PEDOT:PSS solution (left) and blade-coated PEDOT:PSS film (right) from a solution with 5 vol% EG, coated at 1 mm/s, 90°C, and post-processed with methanol/ethanol (4:6), coated at 1 mm/s, 60°C. The respective band between 166 and 171 eV is assigned to the sulfur atom in PSS, and the doublet peaks between 162 and 166 eV correspond to the sulfur atom in the PEDOT benzene ring.

....23

Table of Contents

Section 1. Automated procedures 5
1.1 Film coverage analysis..... 7
1.2 Automated thickness measurements..... 8
1.3 Statistical methods for data repeatability..... 9
1.3.1 Example of a 2-trials sample 9
1.3.2 Example of a 3-trials sample 10
Section 2. Prediction models and autonomous search..... 11
2.1 Initial training data..... 11
2.2 Electrical conductivity predictions 11
2.3 Film defects predictions..... 11
2.4 Bayesian Optimization..... 11
2.5 Importance-guided Bayesian Optimization 12
2.6 UMAP algorithm 13
2.7 Pareto front 13
Section 3. Data analysis..... 15
3.1 Model interpretability using SHAP 15
3.2 Features importance analysis on the training data..... 16
3.3 Binning technique and features importance analysis on all data..... 17
Section 4. In-depth characterizations and large-scale printed films 19
References:..... 21

Section 1. Automated procedures

Supplementary Table 1: Summary of the experimental parameters. The ranges of these parameters are discretized evenly, giving 36 formulations, 90 processing conditions, and 288 post-processing conditions (a total of 933120 possible experimental conditions).

	Parameter name (unit)	Parameter type	Range	Increment	Num. of values
1	DMSO concentration (Vol%)	formulation	0 – 5	1	6
2	EG concentration (Vol%)	formulation	0 – 5	1	6
3	Coating speed (mm/sec)	processing	0.5 – 5	0.5	10
4	Coating temperature (°C)	processing	60 – 100	5	9
5	Post-processing solvent No.	post-processing	1 – 8	1	8
6	Post coating temperature (°C)	post-processing	60 – 100	5	9
7	Post coating speed (mm/sec)	post-processing	0.5 – 2	0.5	4

Supplementary Table 2. Number and Mixing ratios of post-processing solvents.

Post-processing solvent No.	Concentrations (Vol% of MeOH, EtOH, H2O)
1	(100, 0, 0)
2	(20, 80, 0)
3	(40, 60, 0)
4	(50, 50, 0)
5	(60, 40, 0)
6	(80, 20, 0)
7	(0, 100, 0)
8	(80, 0, 20)

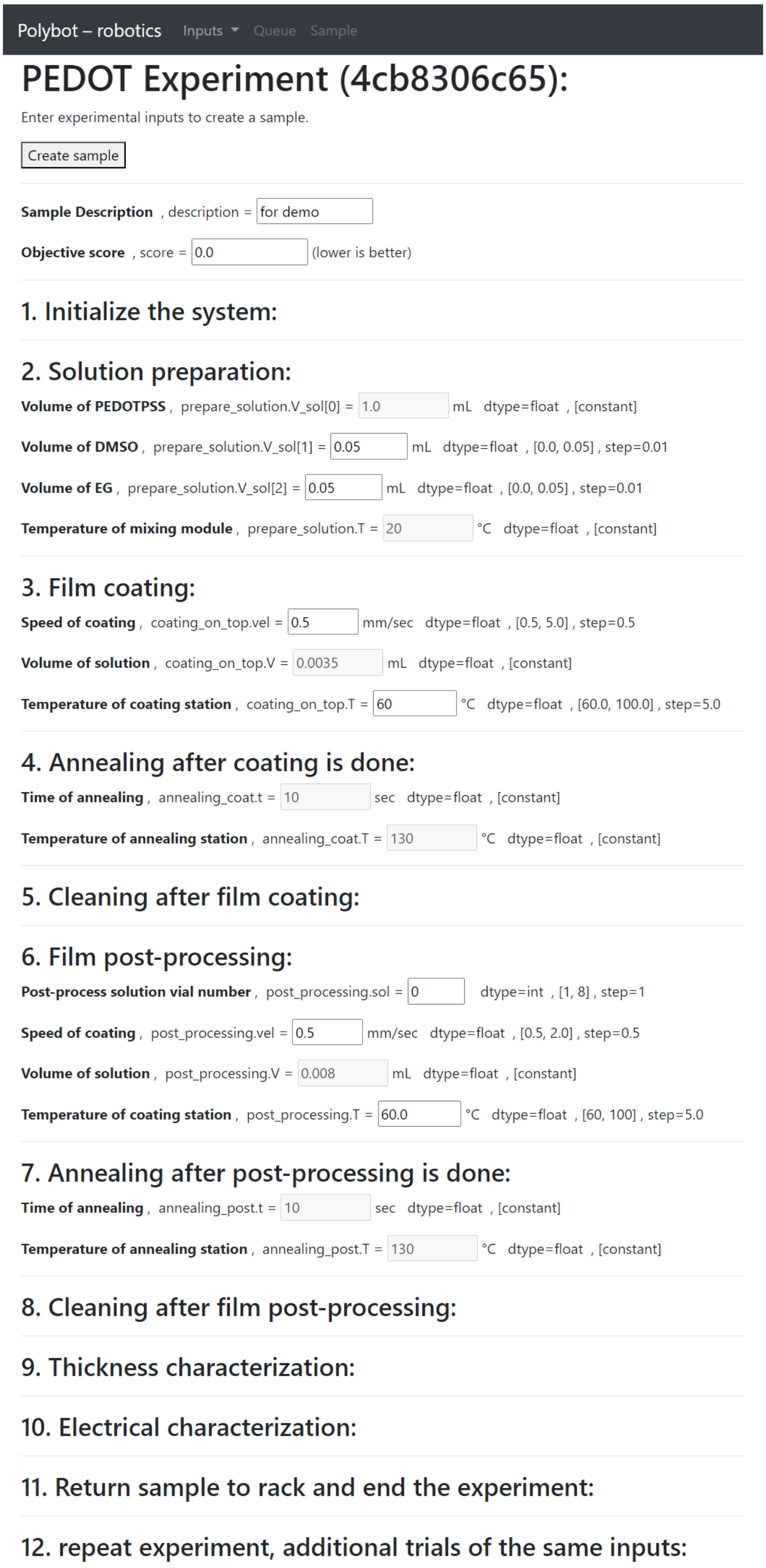

Supplementary Fig. 1. Automated workflow of our thin film experiment. The workflow includes steps in our experimental procedure (described in Section 2) and other additional steps specific to an automated workflow. The graphical user interface allows users to set the experimental parameters and initiate the running of a sample.

1.1 Film coverage analysis

To evaluate the processability of each coating condition, we employ an imaging system (camera) to capture two images: one for substrate-only and another for film coated on the substrate (dried but before annealing). Computer vision algorithms are utilized to extract the film coverage through image analysis. A gray card is employed for white balance corrections. A region of interest (substrate/film occupied pixels) is identified using Harris corner detection followed by geometrical criteria filtering (e.g., side lengths, angles between points, etc.). The hue information from the substrate-only image is utilized to classify pixels in the region of interest in the film-on-substrate image as either substrate or film. The relative number of pixels close to the center (away from edge of the substrate) is used to estimate the percentage of film coverage.

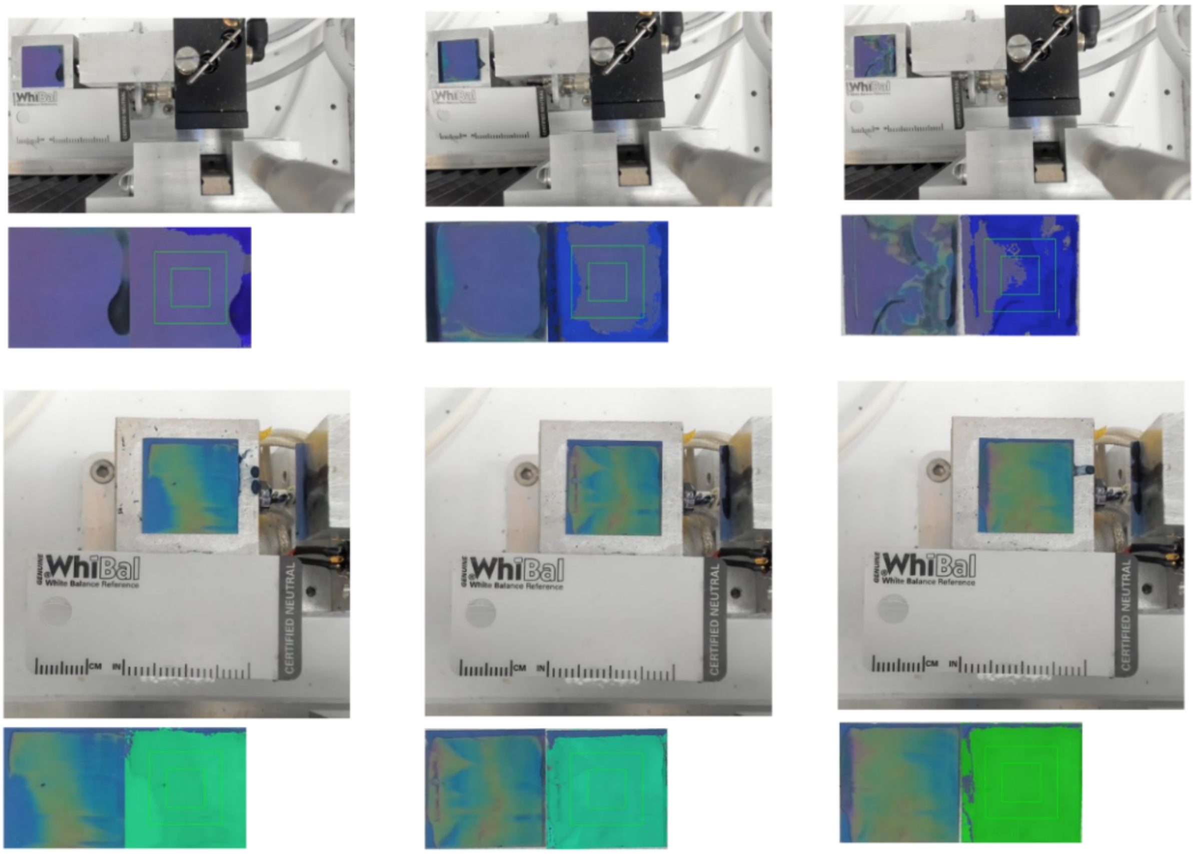

Supplementary Fig. 2. Example images captured by the automated imaging system in Polybot. The images are color corrected using the gray card and processed by computer vision algorithms for film coverage analysis.

1.2 Automated thickness measurements

Due to the hygroscopic nature of PSS, the thickness of PEDOT:PSS thin films is sensitive to the ambient humidity. To ensure inert atmosphere, our robotic system is enclosed and purged with nitrogen (N_2).

Sample raw data:

	Training data 28		Training data 20	
**Measurement location**	**Thickness (nm)**	**R^2 of fitting**	**Thickness (nm)**	**R^2 of fitting**
Top left (point 1)	38.53396	0.9944506	50.17509	0.9927844
Bottom left (point 2)	51.56865	0.9971878	196.1466	0.6948325
Top right (point 3)	31.79736	0.9988059	198.8856	0.8790033
Bottom right (point 4)	28.28061	0.9991889	39.27201	0.9919040

Methods:

- Data with a R^2 of fitting below 0.9 were excluded to maintain analysis reliability.
- Film thickness was averaged at points 1 and 2 for the left side, and points 3 and 4 for the right.
- These averages were then used for a linear interpolation to estimate film thickness at the resistance measurement locations.

	**Training data 28**	**Training data 20**
Left side thickness (nm)	45.051305	50.17509
Right side thickness (nm)	30.038985	39.27201

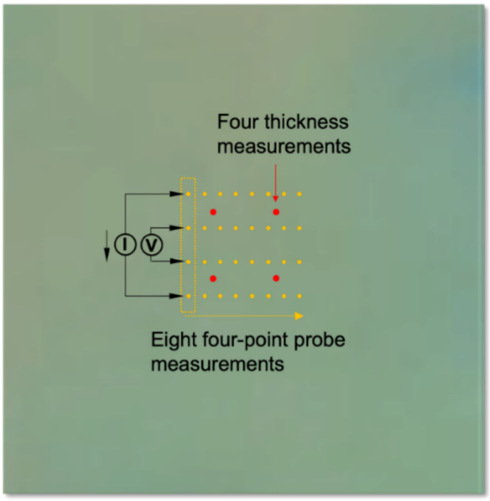

Supplementary Fig. 3. Measurement locations in a sample. The sample size is 2cm x 2cm. Red points mark the locations of four thickness characterizations and yellow points mark the locations of eight four-point probe measurements. The coating direction is from top to bottom.

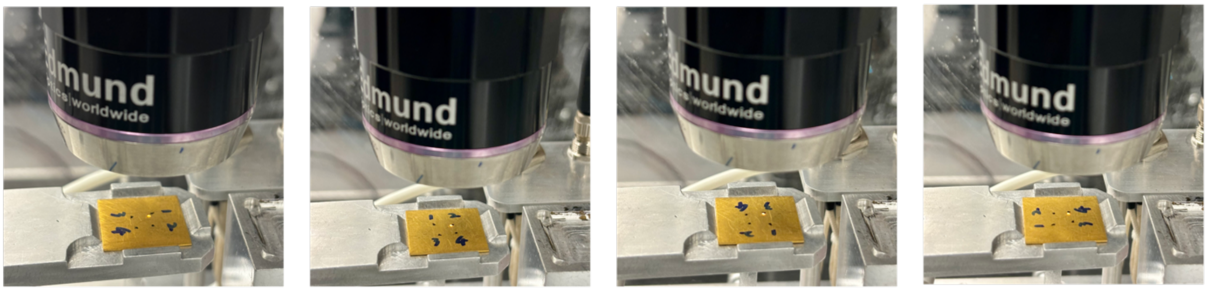

Supplementary Fig. 4. Automated thickness measurement station. The sample is rotated by a robotic gripper for measuring different sample locations.

1.3 Statistical methods for data repeatability

Polybot conducts an experiment multiple times to ensure repeatability. Initially, it gathers all the trials and their results. The results include predicted conductivity and probe coverage percent. Depending on the number of trials (2, 3, or more than 3), the method takes different paths:

For 2 trials, it performs a normality test on each trial. If both trials pass the normality test (indicating a normal distribution), it performs a t-test to compare the means of the two trials. If the p-value from the t-test is larger than the significance level (alpha = 0.005), it averages the two trials. If not, it suggests a third run.

For 3 trials, it first performs a normality test (Shapiro-Wilk test) on each trial. Depending on the number of trials that pass the normality test, it takes different actions. If all trials fail the normality test, it selects the two trials with the closest medians. If only one trial passes the normality test, it suggests a fourth run. If two or three trials pass the normality test, it performs a t-test on the trials that passed the normality test and selects the two trials with the highest p-value. The significance level for the normality test is beta = 0.03.

For more than 3 trials, it first performs a normality test on each trial. If less than two trials pass the normality test, it selects the two trials with the closest medians. If more than two trials pass the normality test, it performs a t-test on the trials that passed the normality test and selects the two trials with the highest p-value.

After selecting the relevant trials, it updates the experiment's results, saves them, and marks the experiment as done.

1.3.1 Example of a 2-trials sample

Measured Data (Test data 2)


```
"all_conductivity_values (S/cm) ": [
        [570.019, 555.975, 537.653, 518.042, 498.188, 476.039, 440.081, 406.362],
        [570.830, 546.915, 525.888, 512.166, 493.990, 478.523, 472.947, 451.465]
 ]
```

Shapiro-Wilk p-value is 0.800 for trial 1 and 0.946 for trial 2, meaning both trials passed the normality test. The t-test p-value is 0.802, which is larger than the significance threshold. Both trials are selected, so a 3rd trial is not triggered.


```
"avg_conductivity (S/cm)": 503.4427737556616
"median_conductivity (S/cm)": 505.17722459278383
```


1.3.2 Example of a 3-trials sample

Measured Data (Test data 12)


```
"all_conductivity_values (S/cm) ": [
        [1097.689, 1206.121, 1332.562, 1269.801, 1497.328, 1457.670, 1357.265, 1171.767],
        [938.233, 898.234, 848.119, 783.923, 761.534, 731.670, 697.792, 679.806],
        [714.010, 707.463, 718.313, 714.642, 716.545, 712.133, 702.761, 711.410]
  ]
```


Shapiro-Wilk p-value is 0.901 for trial 1, 0.625 for trial 2, and 0.658 for trial 3, meaning all trials passed the normality test. The t-test p-value is 6.40E07 for trial 1&2, 1.01E-8 for trial 1&3, and 3.03E-2 for trial 2&3. Only t-test p-value of trial 2&3 is larger than the significance threshold, so trials 2 and 3 are selected.


```
"avg_conductivity (S/cm)": 752.2867092007139,
"median_conductivity (S/cm)": 715.5931520736987,
```

Section 2. Prediction models and autonomous search

2.1 Initial training data

For the initial training data, a limited set of 30 data points is sampled using the Latin Hypercube Sampling (LHS) method. LHS is a quasi-random sampling technique that independently divides the interval associated with each experimental parameter into equally probable bins. Only one sample is drawn from each bin, and samples across all experimental parameters are randomly grouped to produce the required number of training samples. Unlike traditional random sampling, LHS achieves reasonably optimal space-filling of the parameter search space for any number of required samples.

2.2 Electrical conductivity predictions

To predict the average conductivity of thin films, Gaussian processes regression (GPR) is employed as a learning model. Within the Gaussian process, the covariance kernel function is defined by radial basis functions with a length scale of 1 and variance as the square of the averaged conductivity. The prediction model is trained on the initial training data and updated with new data at every iteration of the autonomous search.

2.3 Film defects predictions

To estimate the probability of good thin film processibility (better coverage with less defects), Gaussian Kernel density estimation (KDE) is utilized for the prediction model. Film processibility is primarily quantified through the percentage of film coverage. A 3D grid is constructed based on the ranges and increments of the top three most important experimental processing parameters that influence film coverage, which are identified from the initial training data. This grid is populated with values in the interval [0.1, 0.9], representing the probabilities of choosing the particular processing condition, which is directly proportional to the film coverage. The initial values are set to 0.5 (equal picking, no bias), and subsequent film coverage measurements update these values. The update is done by adding Gaussian kernel with height proportional to the measured film coverage and width set wide enough to mostly affect only the direct neighbouring grid points. Following the updates, the grid is re-normalized to ensure all its values stay in the interval [0.1, 0.9].

2.4 Bayesian Optimization

The Bayesian Optimization method can efficiently navigate the experimental parameter search space, through balancing the exploitation of already collected data and the exploration of uncharted processing conditions. This balance is characterized by the Expected improvement (EI) acquisition function:

$$EI(x) = (\mu(x) - y_{best} - \xi)\, \Phi(Z) + \sigma(x)\, \varphi(Z) \qquad (1)$$

where

$\Phi(Z)$: the cumulative distribution function (CDF) of the standard normal distribution

$\varphi(Z)$: the probability density function (PDF) of the standard normal distribution

$\mu(x)$: predicted mean (average predicted value of the property)

$\sigma(x)$: standard deviation (uncertainty)

y_{best}: the expected best value

ξ: a trade-off parameter that controls exploitation-exploration balance (set to 0.2 in this study, larger ξ favors exploration).

2.5 Importance-guided Bayesian Optimization

Polybot employs a novel optimization strategy for two objectives. To enable autonomous selection of the next experimental conditions. Similar to a traditional Bayesian Optimization, all data points in our experiments are ranked based on EI in conductivity predicted by the GPR model. Normally, the first candidate on the list is the best candidate chosen for the next experiment.

To propose the next experimental conditions, all data points are ranked based on EI in conductivity as predicted by the GPR model, and in the same order until one data point is selected, the data points are considered with a probability that is proportional to the predicted film coverage. In our autonomous experiments, the proposed experimental condition is executed, added to the training data set, and the GPR model is subsequently retrained and improved through an iterative optimization strategy.

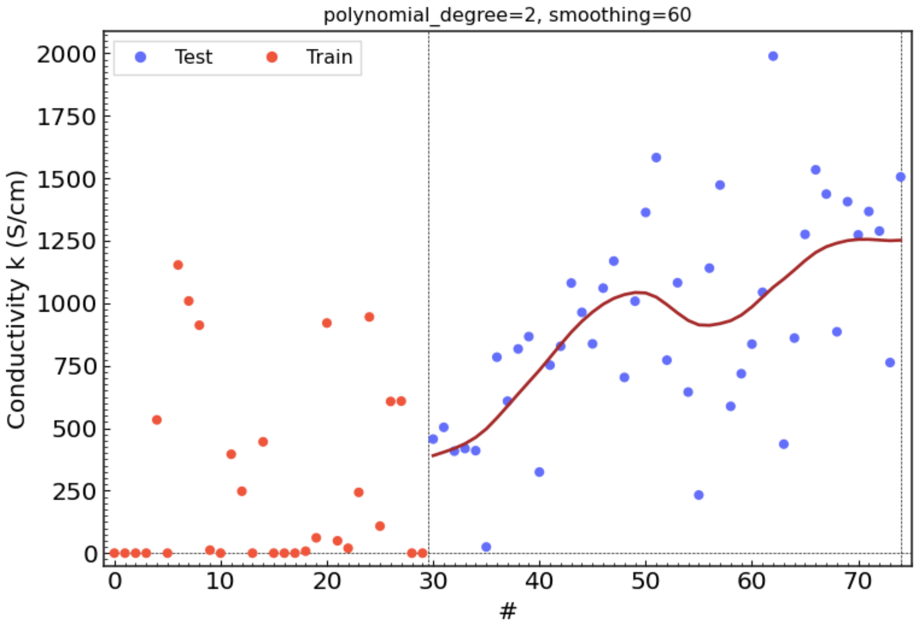

Supplementary Fig. 5. Proof of ML model success. On average, the processing conditions identified by the ML model result in higher conductivity samples (blue circles). The average conductivity in the start from training data is around 300 S/cm. However, at the end of 45 test experiments, the avg. conductivity is 1075 S/cm (more than triple). This proves that the ML model successfully learned what processing conditions result in high conductivity films. After applying a non-monotonic fit method on the test data, it was observed that the resulting curve exhibits a distinct pattern: while it shows variation at the beginning, it eventually transitions into a linear pattern during the final iterations. As such the closed-loop experiment was terminated after reaching this consistent linear pattern.

2.6 UMAP algorithm

UMAP (crystals space Uniform Manifold Approximation and implemented for a low Projection for Dimension Reduction)[1] was dimensional space encoding of the whole experimental dataset. All the input features are normalized to [0,1] to be comparable. The implemented UMAP settings were selected based on the best distance preservation between the high dimensions and the two-dimensional embeddings. The most UMAP settings used are the following: n_neighbours = 5, min_dist = 0.01, Euclidean distance metric and random state=1. We fitted on all the possible experimental space and projected in 2D for both coverage and conductivity.

2.7 Pareto front

The selection of the champion candidates for further scaling up was performed based on the Pareto optimality, which is a commonly used criterion for determining solutions to multi-objective optimization problems. We selected four points from the Pareto frontier with the best trade-off between conductivity and coverage.

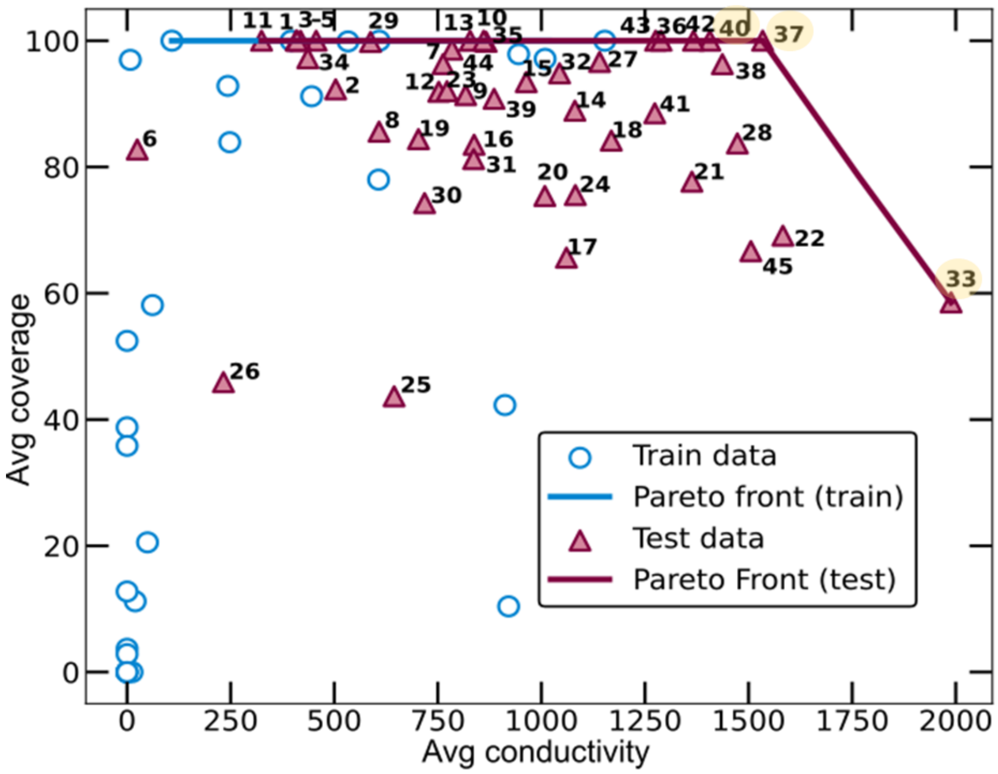

Supplementary Fig. 6. Scatterplot showing the trade-off between average conductivity (S/cm) and average coverage (%) for both the training and test data. Only the test datapoints are labelled in the plots and the champion candidates for further exploration are highlighted in yellow.

Supplementary Table 3. Actual values of the processing parameters for the three selected champion candidates.

	Test data 33	Test data 37	Test data 40
Sample ID	86370bcb61	bd92c41523	0e7b6469cf
Parameter name (unit)			
DMSO concentration (Vol%)	0	0	0
EG concentration (Vol%)	5	5	5
Coating speed (mm/sec)	1	1	1
Coating temperature (°C)	90	95	95
Post-processing solvent No.<br>(Vol% of MeOH, EtOH, H2O)	3<br>(40, 60, 0)	5<br>(60, 40, 0)	6<br>(80, 20, 0)
Post coating temperature (°C)	60	75	70
Post coating speed (mm/sec)	1.0	1.0	1.0
Measured performance (unit)			
Actual conductivity (S/cm)	1988.672	1533.952	1406.392
Actual conductivity std (S/cm)	3993.827	292.448	318.773
Actual coverage (%)	58.6	100.0	100.0
Actual coverage std (%)	15.3	0.0	0.0

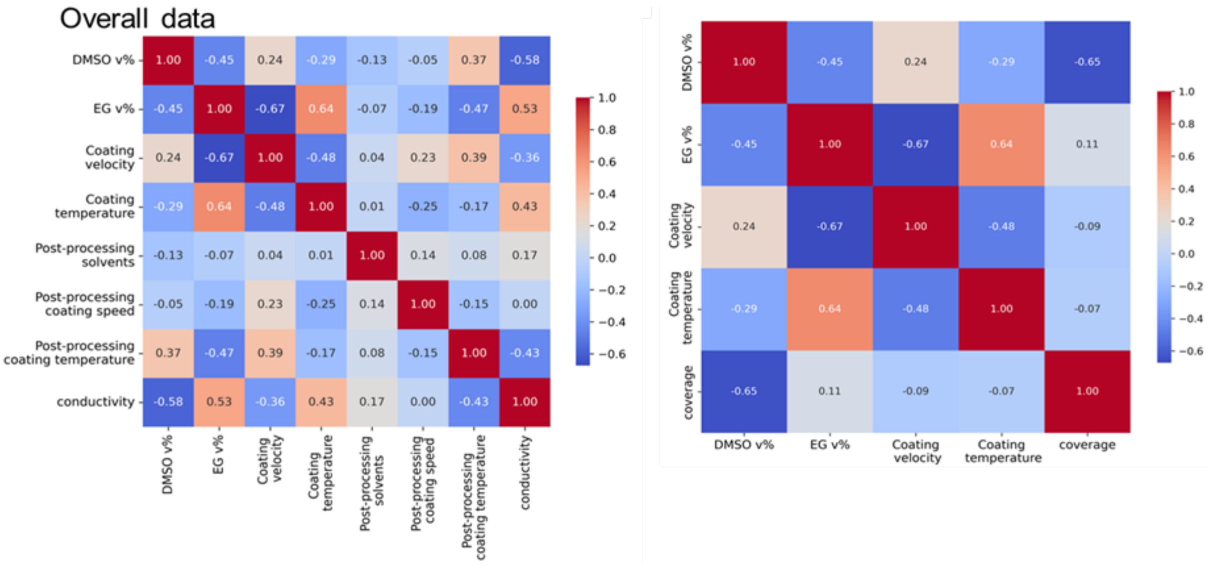

Supplementary Fig. 7. Correlation matrices for conductivity (left) and coverage (right) on the final dataset acquired from the autonomous experiment.

Section 3. Data analysis

To derive insights into the factors influencing film performance, we initially apply ML models to the dataset and compare their performance. Following the evaluation of these models, we select the optimal model to conduct Shapley analysis (see section 5.1 below), enabling us to determine the feature impact. The procedure is applied on the training data only and on the combined train and test data after the completion of the autonomous experiment. As coverage is measured before the post-processing step, given the minimal observed influence of post-processing observed on coverage, only formulation and processing conditions are considered for coverage prediction model. Conversely, all formulation, processing, and post-processing factors are taken into account for predicting conductivity.

3.1 Model interpretability using SHAP

SHAP (Shapley Additive exPlanations), an approach derived from coorporative game theory, is implemented as a model interpretation framework for providing insights into this machine learning predictions. SHAP is a model independent method, meaning that it is not takes into consideration the feature weights but measures the influence each feature change has on the final decision of the model. In other words, by calculating Shapley values, the contribution of each feature of each combination to the final score is estimated. The overall SHAP formula is shown in equation (1), where g is the explanation model, M is the number of simplified input features, $\varphi i \in \mathbb{R}$ is the feature attribution for a feature i, $z' \in \{0,1\}\ M$, and $\varphi 0$ represents the model output with all the simplified inputs missing.

$$g(z')=\varphi 0+\sum \varphi i z' i M \iota=1 \qquad (2)$$

To obtain the contribution of a feature i, all operations by which a feature might have been added to the set (N!) and a summation over all possible sets (S) is considered. For any feature sequence, the marginal contribution through addition of feature i is given by $[f(S \cup \{i\}) - f(S)]$, where $f(S)$ corresponds to the output of the ML model. The resulting quantity is weighted by the different possibilities the set could have been formed prior to feature i's addition (|S|!) and the remaining features could have been added ((|N| − |S| − 1)!). Hence, the importance of a given feature is defined by equation (2):

$$\varphi i=1\ N!\ \sum |S|!(|N|-|S|-1)![f(S\cup\{i\})-f(S)]S\subseteq \mathbf{N}\backslash\{\mathbf{i}\} \qquad (3)$$

It follows that Shapley values represent a unique way to divide a model's output among feature contributions satisfying three axioms: local accuracy (or additivity), consistency (or symmetry), and nonexistence (or null effect). Using the SHAP approach, the identification and prioritization of features that determine the pairs ranking is enabled. In that way we can extract the connection between the process parameters and conductivity and coverage. In addition to model accuracy, the interpretability of the predictions is adding value to any machine learning model. High negative Shapley values are driving the model towards low values of the property of interest, whereas as high positive values are contributing towards high property values. The Tree Explainer method was used to calculate the contribution of the parameters towards conductivity and coverage.

3.2 Features importance analysis on the training data

Supplementary Table 4. Machine learning regression models for coverage prediction on the train data only. A random seed of 1 was used in all the cases to achieve repeatability.

ML models	Input variables	Hyperparameters	R^2
Linear regression	-DMSO concentration (Vol%)<br>-EG concentration (Vol%)<br>-Coating speed (mm/sec)<br>-Coating temperature (°C)		0.59
kNeighbors Regressor		n_neighbors = 5	0.70
Support Vector Regressor		kernel = “rbf”, C=100, gamma=”auto”, epsilon=0.1	0.56
Random forest regressor		max_depth=8	0.76
AutoML			0.58

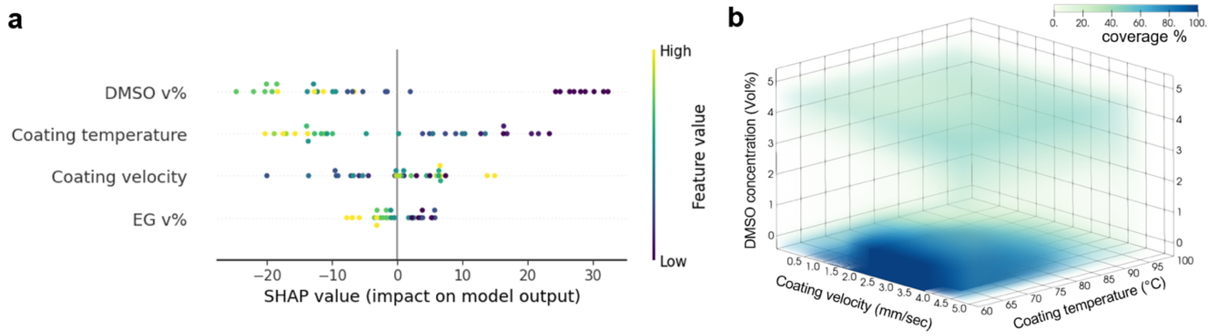

Supplementary Fig. 8. a) Shapley feature importance showing the effect of the various parameters in coverage after fitting the best performing Random Forest model on the train data. We can clearly see that the top three important features are the DMSO%, coating temperature with a negative impact to coverage and coating speed with a positive impact in coverage. b) Density mapping of the most important parameters that affect the coverage as observed.

Supplementary Table 5. Machine learning regression models for predicting conductivity using training data only. A random seed of 1 was used in all the cases to achieve repeatability. Given the uneven distribution of the target property, achieving predictive accuracy is challenging due to the significantly imbalanced data.

ML models	Input variables	Hyperparameters	R^2
Linear regression	-DMSO concentration (Vol%)<br>-EG concentration (Vol%)<br>-Coating speed (mm/sec)<br>-Coating temperature (°C)<br>-Post-processing solvent No.<br>-Post coating temperature (°C)<br>-Post coating speed (mm/sec)		-0.41
kNeighbors Regressor		n_neighbors = 5	0.01
Support Vector Regressor		kernel = “rbf”, C=100, gamma=”auto”, epsilon=0.1	-0.36
Random forest regressor		max_depth=8	-0.78
AutoML			-0.24

3.3 Binning technique and features importance analysis on all data

One important issue that arise in autonomous experiments is that as the objective is to achieve a desired property value, the final dataset might not have a normal distribution of the property of interest making it challenging to effectively fit an ML model. For that reason, we used a binning technique to split the datapoints in bins with equal amount of data and then used stratified splitting when fitting the ML models.

From the histogram we can calculate the bin edges:

```
hist, bin_edges = np.histogram(pedot_data['conduct_actual'], bins='fd')
```

Then the bin edges are used to categorize the data into classes

```
def map_float_to_class(number):
  if bin_edges[0]<=number<bin_edges[1]:
    return 0
  if bin_edges[1]<=number<bin_edges[2]:
    return 1
  if bin_edges[2]<=number:<bin_edges[3]:
    return 2
```

As such all the datapoints are categorized based on the value of average conductivity into three classes. When the train/test split is performed to train and evaluate the ML algorithms, stratified splitting is applied as such there will be a balance in the average conductivity values.

Supplementary Table 6. ML models for coverage predictions. For each model type, three separate models are trained using: training data only, test data only, and all data (training + test data). The random seed parameter was set to 1 for repeatability.

ML models	Input variables	Hyperparameters	R^2
Linear regression	- DMSO concentration (Vol%)<br>- EG concentration (Vol%)<br>- Coating speed (mm/sec)<br>- Coating temperature (°C)		0.56
kNeighbors Regressor		n_neighbors = 5	0.74
Support Vector Regressor		kernel = “rbf”, C=100, gamma=”auto”, epsilon=0.1	0.75
Random forest regressor		max_depth=8	0.77
AutoML			0.58

Supplementary Table 7. ML models for conductivity predictions.

For each model type, three separate models are trained using: training data only, test data only, and all data (training + test data). The random seed parameter was set to 1 for repeatability.

ML models	Input variables	Hyperparameters	R^2
Linear regression	-DMSO concentration (Vol%)<br>-EG concentration (Vol%)<br>-Coating speed (mm/sec)<br>-Coating temperature (°C)<br>-Post-processing solvent No.<br>-Post coating temperature (°C)<br>-Post coating speed (mm/sec)		0.50
kNeighbors Regressor		n_neighbors = 5	0.58
Support Vector Regressor		kernel = "rbf", C=100, gamma="auto", epsilon=0.1	0.70
Random forest regressor		max_depth=8	0.78
AutoML			0.69

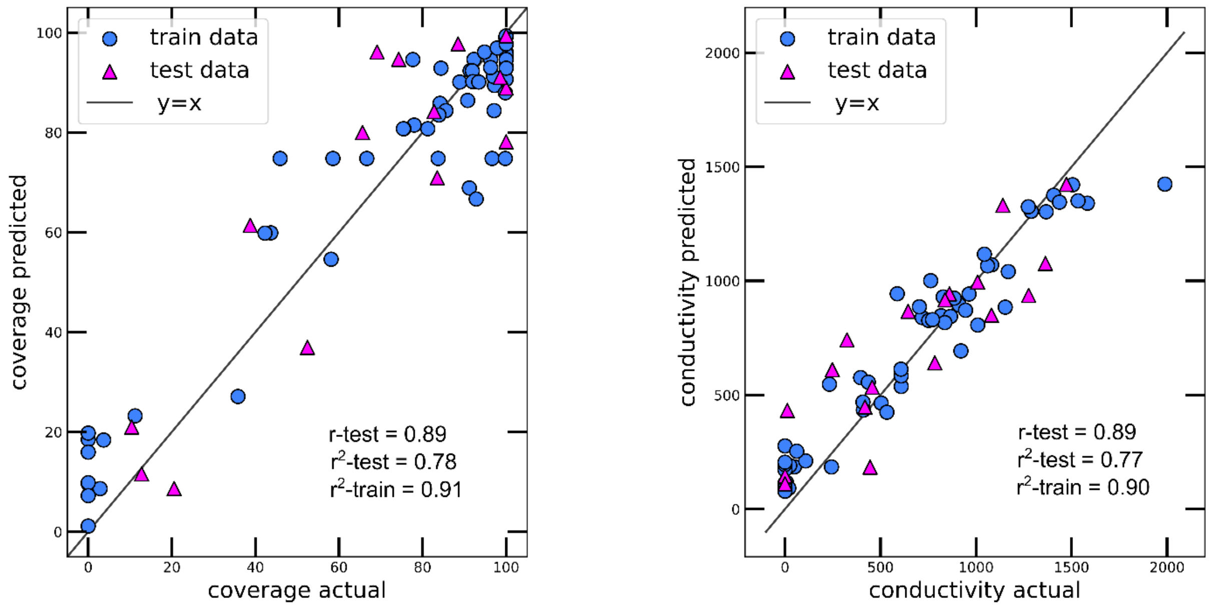

Supplementary Fig. 9. Parity plots for the coverage (%) and conductivity (S/cm) predictions. Left) Predicted versus actual coverage after fitting the best performing model in the collected datapoints. Right) Predicted versus actual average conductivity after fitting the best performing model in all the collected data points.

Section 4. In-depth characterizations and large-scale printed films

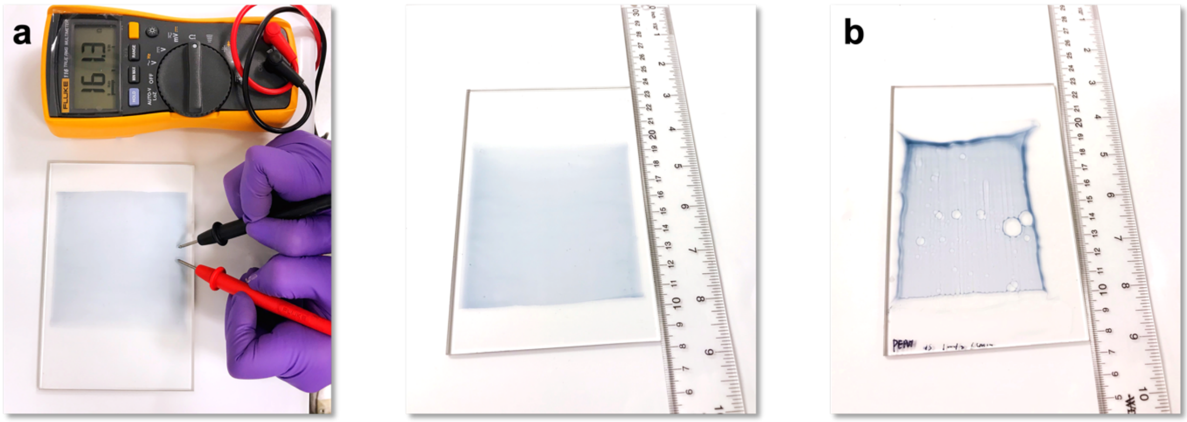

Supplementary Fig. 10. Large PEDOT:PSS conductive thin films prepared using blade coating. a). PEDOT:PSS conductive thin film blade-coated with one of the best conditions (test 33 in Supplementary Table 3) identified by Polybot. b) PEDOT:PSS thin film blade-coated using the identical processing conditions, but using 2 vol% DMSO as the additive in the formulation.

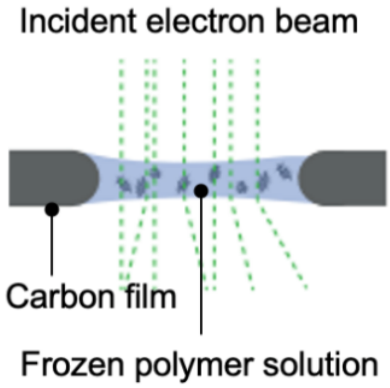

Supplementary Fig. 11. The working principle of cryo-EM on frozen polymer solution. The cryo-EM samples were prepared by a Thermo Scientific Vitrobot Mark IV System. The samples were loaded to a FISCHIONE 2550 cryo transfer tomography holder for imaging on a FEI Talos 200FX (S)TEM operated at 200kV.

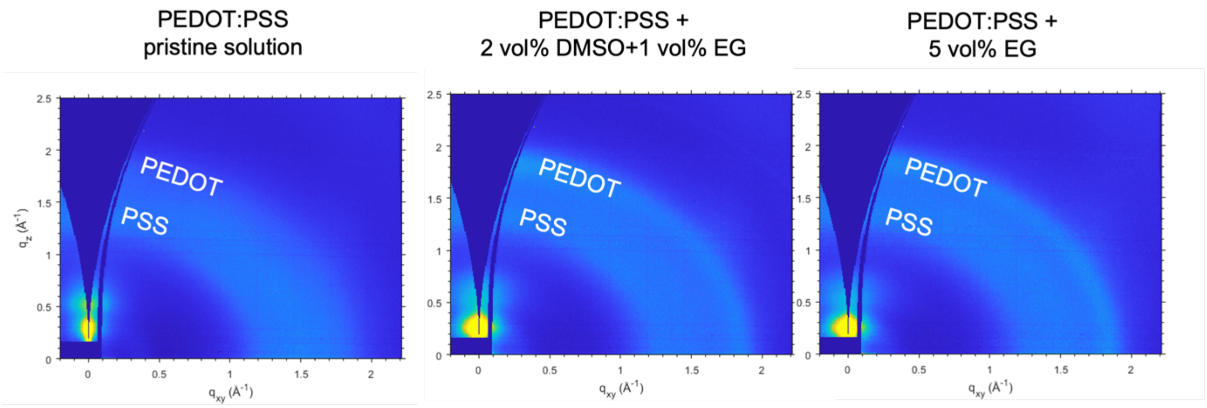

Supplementary Fig. 12. GIWAXS 2D images of three PEDOT:PSS thin films. Left: Spin-coated film from pristine PEDOT:PSS solution (control). Middle: Blade-coated PEDOT:PSS film from a solution with 2 vol% DMSO and 1 vol% EG, coated at 3 mm/s, 60°C, and post-processed with methanol/ethanol (1:1), coated at 3 mm/s, 70°C. Right: Blade-coated PEDOT:PSS film from a solution with 5 vol% EG, coated at 1 mm/s, 90°C, and post-processed with methanol/ethanol (4:6), coated at 1 mm/s, 60°C. GIWAXS measurements were performed at beamline 11-BM of National Synchrotron Light Source II, Brookhaven National Laboratory. The samples were tilted at incident angle of 0.12° with respect to incident beam. All images were collected under vacuum with an incident beam energy of 13.5 keV and calibrated with silver behenate. For images taken, the area detector was translated vertically for a second exposure. The two images were combined to eliminate gaps due to rows of inactive pixels at the borders between modules.

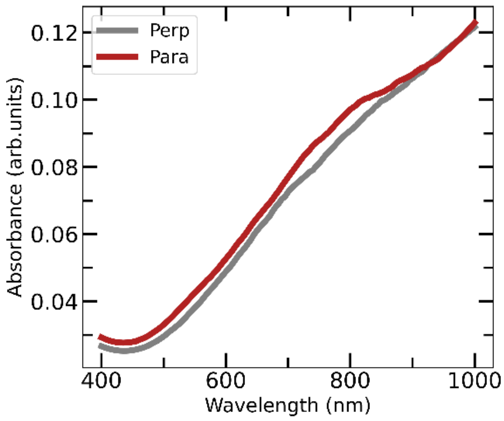

Supplementary Fig. 13. Polarized ultraviolet–visible absorption spectra of blade-coated PEDOT:PSS film from a solution with 5 vol% EG, coated at 1 mm/s, 90°C, and post-processed with methanol/ethanol (4:6), coated at 1 mm/s, 60°C. The red (Para) curve was obtained when the film's coating direction was aligned with the polarizer axis, whereas the gray curve (Perp) was collected with the coating direction perpendicular to the polarizer axis.

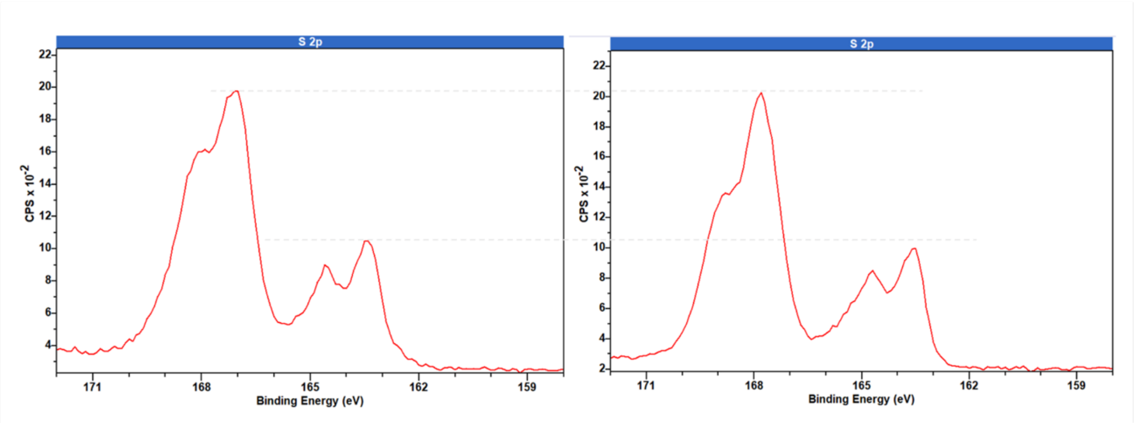

Supplementary Fig. 14. *S (2p)* X-ray photoelectron spectra of spin-coated film from pristine PEDOT:PSS solution (left) and blade-coated PEDOT:PSS film (right) from a solution with 5 vol% EG, coated at 1 mm/s, 90°C, and post-processed with methanol/ethanol (4:6), coated at 1 mm/s, 60°C. The respective band between 166 and 171 eV is assigned to the sulfur atom in PSS, and the doublet peaks between 162 and 166 eV correspond to the sulfur atom in the PEDOT benzene ring. The X-ray photoelectron spectroscopy (XPS) was done with Kratos AXIS Nova with a monochromatic Al Kα X-ray source and a delay line detector (DLD) system.

References:

1. McInnes, L., Healy, J. & Melville, J. UMAP: Uniform Manifold Approximation and Projection for Dimension Reduction. (2018) doi:10.48550/arxiv.1802.03426
