# Supplementary material for: Autonomous platform for solution processing of electronic polymers
Source: Nat Commun. 2025 Feb 17;16:1498. doi: 10.1038/s41467-024-55655-3 (PMC11833048; doi:10.1038/s41467-024-55655-3)
Supplement: Supplementary file 3 — Description of Additional Supplementary Files [file 41467_2024_55655_MOESM3_ESM.pdf]

Description of Additional Supplementary Files

Supplementary Movie 1. One automated workflow within the closed-loop exploration process. The video is accelerated by 4 times

Supplementary Movie 2. Scale-up blade-coating process of highconductivity and low-defect PEDOT:PSS film utilizing the best recipe discovered through autonomous experiments

Supplementary Movie 3. Roll-to-roll coating process of high-conductivity and low-defect PEDOT:PSS film utilizing the best recipe discovered through autonomous experiments

Supplementary Movie 4. Scale-up blade-coating process of PEDOT:PSS film from solution with 2 vol% DMSO.
